# Rapid, novel screening of toxicants in poison baits, and autopsy specimens by ambient mass spectrometry

**DOI:** 10.3389/fchem.2022.982377

**Published:** 2022-08-25

**Authors:** Alessandra Tata, Ivana Pallante, Carmela Zacometti, Alessandra Moressa, Marco Bragolusi, Alessandro Negro, Andrea Massaro, Giovanni Binato, Federica Gallocchio, Roberto Angeletti, Nicola Pozzato, Roberto Piro

**Affiliations:** ^1^ Istituto Zooprofilattico Sperimentale delle Venezie, Laboratorio di Chimica Sperimentale, Vicenza, Italy; ^2^ Istituto Zooprofilattico Sperimentale delle Venezie, Laboratorio di Medicina Forense Veterinaria, Vicenza, Italy; ^3^ Istituto Zooprofilattico Sperimentale delle Venezie, Chimica, Legnaro, Italy

**Keywords:** DART-HRMS, QuEChERS, bitter agent, suspected animal poisoning, toxicology

## Abstract

Animal poisoning and dissemination of baits in the environment have public health and ethological implications, which can be followed by criminal sanctions for those responsible. The reference methods for the analysis of suspect baits and autopsy specimens are founded on chromatographic-based techniques. They are extremely robust and sensitive, but also very expensive and laborious. For this reason, we developed an ambient mass spectrometry (AMS) method able to screen for 40 toxicants including carbamates, organophosphate and chlorinated pesticides, coumarins, metaldehyde, and strychnine. Spiked samples were firstly purified and extracted by dispersive solid phase extraction (QuEChERS) and then analyzed by direct analysis in real time high-resolution mass spectrometry (DART-HRMS). To verify the performance of this new approach, 115 authentic baits (*n* = 59) and necropsy specimens (gastrointestinal content and liver, *n* = 56) were assessed by the official reference methods and combined QuEChERS-DART-HRMS. The agreement between the results allowed evaluation of the performances of the new screening method for a variety of analytes and calculation of the resultant statistical indicators (the new method had overall accuracy 89.57%, sensitivity of 88.24%, and a specificity of 91.49%). Taking into account only the baits, 96.61% of overall accuracy was achieved with 57/59 samples correctly identified (statistical sensitivity 97.50%, statistical specificity 94.74%). Successful identification of the bitter compound, denatonium benzoate, in all the samples that contained rodenticides (28/28) was also achieved. We believe initial screening of suspect poison baits could guide the choice of reference confirmatory methods, reduce the load in official laboratories, and help the early stages of investigations into cases of animal poisoning.

## Introduction

Intentional animal poisoning can cause unintentional damage to the environment and animal and human health (Bille et al., 2016). However, intentional poisoning is widely practiced around the world and mainly targets dogs and cats, but also other domestic and wild animals (Berny et al., 2010; Caloni et al., 2012, Guitart et al., 2022). This phenomenon is a problem that concerns public health authorities, because it is not rare that people, especially children, are also victims of intentional animal poisoning (De Rom et el 2018). Since 2008, the Italian Ministry of Health issued some ordinances regarding the “Rules on the Prohibition of Use and Detention of Baits and or Poison baits” with the aim to reduce intentional poisoning incidents. In 2019, an informatics platform, named “Portale Nazionale degli avvelenamenti dolosi degli animali,” was created, which notifies the Italian public veterinary health institutions of intentional poisoning episodes in animals and the illegal use of poison baits. Although several measures have been adopted to counter the poisoning phenomenon, no reduction in the frequency of animal poisonings has been recorded in Italy (Muscarella et al., 2016, Chiari et al., 2017). In accordance with ordinance issued by the Italian Ministry of Health, toxicological investigations are mandatory and executed by Istituti Zooprofilattici Sperimentali (IIZZSS) (Ministero della Salute, Rome, 2021).

The toxic compounds (which are also analytes) that are mainly used in these poisoning events are carbamates, organophosphate and chlorinated pesticides, coumaric rodenticides, metaldehyde, strychnine, and ethylene glycol. The management of rats mostly relies on use of coumaric rodenticides, which are unsafe for human beings, pets and other non-targets. For this reason, denatonium benzoate, a bitter and non-toxic compound, is always added to wax formulations together with the rodenticides.

Different analytical approaches have been proposed in the last 10 years to confirm suspected poisonings. In 2008 Vandenbroucke et al. validated a multi-residual liquid chromatography mass spectrometry (LC-MS) method for the quantitative determination of eight anticoagulant rodenticides in biological matrices like plasma and liver (Vandenbroucke et al., 2008). A LC-MS method that involved the direct analysis of crude ethyl acetate extracts from different specimens taken from animals suspected as being victims of accidental or intentional exposure to pesticides was published in 2013 (Taylor et al., 2013). One year later, a multi-residual approach for the identification of pesticides involved in the poisoning of wild animals using the combination of gas-chromatography mass spectrometry (GC-MS) and LC-MS was proposed by Luzardo et al. (2014). According to Valverde et al. (2018), GC-MS is the most commonly used analytical technique for pesticides, while LC-MS is the main technique to analyze anticoagulant rodenticides . Conversely, last year Gallocchio et al. (2021) successfully developed a LC-MS method for the simultaneous analysis of 13 carbamate pesticides and 8 anticoagulant rodenticides, and successfully tested it on hundreds of authentic samples .

In Italy, the official approach to detect toxins in baits and autopsy specimens is based on multiple chromatographic techniques. Although these analyses are highly sensitive and accurate, they require long times to be executed and for the results to be available.

In this study, we used the combination of the QuEChERS (quick, easy, cheap, effective, rugged, and safe) approach with direct analysis in real time high resolution mass spectrometry (DART-HRMS). This combined method was set up with the aim of screening autopsy specimens (liver and gastrointestinal contents) and baits, thus speeding up and guiding the choice of toxicological reference methods. The samples were both screened by DART-HRMS and examined by reference methods. DART-HRMS is characterized by the direct introduction of samples at ambient conditions into the mass spectrometer without prior chromatographic separation (Gross, 2014). Among multiple applications, it was successfully employed in forensic toxicology (Musah et al., 2012; Duvivier et al., 2014; Chen et al., 2016; Coon et al., 2019; Longo and Musah et al., 2020; Pozzato et al., 2020; Chambers and Musah, 2022), food spoilage (Massaro et al., 2021a; Massaro et al., 2021b; Tata et al., 2022a), and drug analysis (Schurek et al., 2008). In the present study, we captured the presence of 40 different toxicants and one bitter compound (denatonium benzoate) in autopsy specimens and baits. The method was tested on 115 authentic samples from suspected cases of animal poisoning and the results compared with those of official methods.

## Materials and methods

### Certified analytical standards

The instrumental parameters were optimized using certified analytical standards. A mixture of 10 coumarins (brodifacoum, bromadiolone, coumachlor, coumafuryl, coumatetralyl, difenacoum, diphenadione, flocoumafen, pindone, warfarin) were purchased from Dr. Ehrenstofer GmbH (Augsburg, Germany) at concentrations of 500 mg/L in acetonitrile. Certified analytical mixture of 13 carbamates (aldicarb, bendiocarb, benfuracarb, carbaryl, carbofuran, carbosulfan, ethiofencarb, furathiocarb, methiocarb, methomyl, oxamyl, pirimicarb, propoxur) (500 mg/L of each in acetonitrile) was supplied by Restek (Bellefonte, PA, United States). A certified mixture of eight pesticides contained azinphos-ethyl, disulfoton, malathion, parathion, parathion-methyl, phosmet, phoxim, and profenofos (Dr. Ehrenstofer GmbH (Augsburg, Germany). A certified mixture of six pyrethroid pesticides (permethrin, cypermethrin, fenvalerate, cyfluthrin, cyhalothrin, deltamethrin) was purchased from Dr. Ehrenstofer GmbH (Augsburg, Germany). No certified materials were available in our laboratory for endosulfan, metaldehyde, methamidophos or denatonium benzoate.

### Samples

Spiked homogenized sausages with 100 μg/kg, 500 μg/kg and 5,000 μg/kg of toxic analytes were used to verify the detection limit (LOD) of the method for each analyte. Moreover, a total of 115 autopsy specimens and baits (56 autopsy specimens and 59 baits) found in cases of suspected animal poisoning were analyzed both by the novel QuEChERS-DART-HRMS method and reference methods in order to verify the performance of the screening method. These authentic samples were used to validate the performances of the technique for a variety of relevant analytes.

### Reference methods

Two official reference methods were carried out on the 115 samples (autopsy specimens and baits) found in cases of suspected animal poisoning as described in other previously published studies from our group (Gallocchio; Moressa; Stella; Rosin *Et Al.*, 2021) (Bille; Toson; Mulatti; Dalla Pozza Et Al., 2016). Briefly, sample extracts were analyzed by GC-MS (GCMS-QP2010 Plus Shimadzu, Kyoto, Japan) in full scan mode to detect GC amenable compounds (azinphos-ethyl, disulfoton, malathion, parathion, parathion-methyl, phosmet, phoxim, profenofos, permethrin, cypermethrin, fenvalerate, cyfluthrin, cyhalothrin, deltamethrin endosulfan, metaldehyde, methamidophos) (Bille; Toson; Mulatti; Dalla Pozza Et Al., 2016). These extracts were diluted 10-fold with MilliQ water and analyzed by the UHPLC-ESI-orbitrap-MS system (Thermo Fisher Scientific, Bremen, Germany), in full scan and data-dependent MS/MS fragmentation spectra mode, to detect LC amenable compounds (brodifacoum, bromadiolone, coumachlor, coumafuryl, coumatetralyl, difenacoum, flocoumafen, warfarin, aldicarb, bendiocarb, benfuracarb, carbaryl, carbofuran, carbosulfan, ethiofencarb, furathiocarb, methiocarb, methomyl, oxamyl, pirimicarb, and propoxur) (Gallocchio; Moressa; Stella; Rosin Et Al., 2021). The limit of quantification (LOQ) was 100 μg/kg for both GC-MS and UHPLC-ESI-orbitrap-MS analysis.

### Sample preparation by QuEChERS prior to both DART-HRMS and analysis by reference methods

An amount of 2 ± 0.1 g of each homogenized sample was suspended in 20 ml of water/acetonitrile (50:50, v/v), mixed with QuEChERS salts (4 g Na_2_SO_4_, 1 g NaCl, 1 g trisodium citrate dehydrate, and 0.5 g disodium hydrogen citrate sesquihydrate), vortexed for 1 min and centrifuged for 5 min at 3,900 × g. Afterwards, the extracts were cleaned up by dispersive solid phase extraction (dSPE). To this aim, 6 ml of extract was transferred into plastic tube containing 150 mg of primary and secondary amine exchange material (PSA), 150 mg of C18 powder, and 900 mg MgSO_4_, vortexed and centrifuged for 5 min at 3,900 × g. Supernatant (1 ml) was again decanted and centrifuged, this time at 12,000 × g for 5 min. The final supernatant (hereafter called the sample extract) was analyzed by GC-MS, LC-MS (reference method analysis) and DART-HRMS. The QuEChERS and SPE kits were purchased from Agilent Technologies (Santa Clara, CA, United States).

### DART-HRMS analysis

Ambient mass spectrometry was carried out using a DART SVP 100 ion source (IonSense, Saugus, MA, United States) coupled to an Exactive Orbitrap (Thermo Fisher Scientific, Waltham, MA, United States). A 5 µl volume of the sample extract was placed on a glass capillary rod. A Dip-it^(R)^ autosampler allowed the automatic positioning of the glass capillary rod in front of the source (IonSense, Saugus, MA, United States). The optimized DART settings for analysis of the carbamates were set as follows: grid voltage, 100 V; helium flow, 4.26 L/min; temperature, 100°C; sample speed, 0.3 mm/s; single time analysis, 0.66 min. Settings of the parameters of the mass spectrometer were as follows: S-lens RF level, 55; capillary temperature, 100°C; maximum injection time, 10 ms. The optimized DART settings for the analysis of the pyrethroids, coumarins, metaldehyde, strychnine, and organophosphate and chlorinated pesticides were set as follows: grid voltage, 100 V; helium flow, 4.26 L/min; temperature, 350°C; sample speed 0.3 mm/s; single time analysis, 0.66 min. Settings of the parameters of the mass spectrometer were as follows: S-lens RF level, 55; capillary temperature, 350°C; maximum injection time, 10 ms. The resolution was set to 70,000 full width at half maximum (FWHM) and the mass range was 75–1,125 Da in positive ion mode. Although a few toxic analytes could be better ionized in negative ion mode (e.i., endosulfan), positive ion mode (+DART) was preferred as the majority of the compounds were expected to ionize well in this polarity. Each sample extract was analyzed in triplicate to determine repeatability.

### Targeted analysis

Skyline software (https://skyline.ms/project/home/software/Skyline/begin.view) was used to interrogate the data on the targeted molecules. Skyline is a freely-available and open source application that has been used for analyzing large-scale, raw mass spectra and building customized interrogation assays. In detail, we created two customized targeted methods (one for coumarin-strychnine-denatonium and one for carbamates-pyretroids-organophosphate-organochlorurate-metaldeyde-endosulfan) able to select and refine target ion peaks in the time range 0.1–0.4 min (which is the interval in which the DART-HRMS total ion current is maximized), with a minimum of peak area of 1 × 10^6^ and a maximum mass error of 5 ppm.

After exporting the so-called transition list of the results from Skyline, a R script, created by our statistician, was able to generate a final table in Excel (Microsoft) with a list of analyzed samples and the related detected molecules.

## Results

Initially, QuEChERS was used for the clean-up and extraction of toxicants from spiked samples. The Supplementary Figure S1 of the supplementary material shows how the DART-HRMS signal of toxicants can be enhanced when QuEChERS clean-up is applied (as compared to simple acetonitrile liquid extraction). To determine the LOD of the toxic compounds analyzed by the novel QuEChERS-DART-HRMS method, we prepared spiked sausages with concentrations of 100 μg/kg, 500 μg/kg, and 5,000 μg/kg. While the minimum concentration at which the majority of the analytes were detected was 500 μg/kg, four organophosphates (disulfoton, parathion, parathion-methyl, and phoxim) were detected at the higher LOD, 5 mg/kg (Supplementary Table S1).

Intra-sample repeatability was assessed by CV% as an indicator of the instrument intraday fluctuations. To this aim, the signal intensities of the analytes in spiked samples at the LOD level and at 5,000 μg/kg were monitored during the repeated DART-HRMS data acquisition (triplicate) (Supplementary Table S1). The resulting CV% at the LOD level was <20% for 31 out of 37 analytes, showing the method is characterized by a small degree of variation and, thus, high precision. Poor CV% values were observed for 6 out of 37 analytes. At the higher concentration (10 times higher) the CV% values were lower, with 36/37 analytes presenting a CV% of <20%.

Using the proposed experimental set-up at a concentration of 5,000 μg/kg, recoveries from almost 15% up to 40% were achieved for most of the toxic compounds in spiked samples (Supplementary Table S2 of SI). Lower recoveries were observed for carbosulfan, brodifacoum, difenacoum, phoxim, parathion, parathion-methyl, permethrin, and strychnine (Supplementary Table S2). However, the detection of these compounds was still possible by DART-HRMS.

Analytical specificity was assessed in blank samples (homogenized sausages), by verifying the absence of the corresponding *m/z* of each toxicant (within the 5 ppm error) higher than 30% of the LOD.

As an example, the overlapped spectra of methamidophos detected in the spiked material and in a real autoptic specimen are reported in Supplementary Figure S2. To verify the actual performances of the novel screening method for each analyte in authentic samples, 115 samples from suspected poisoning cases were analyzed both by the official reference methods and QuEChERS extraction combined with DART-HRMS. Table 1 reports the analytes detected in each authentic sample, according to both QuEChERS-DART-HRMS and reference methods. Table 2 lists the other compounds detected in the QuEChERS-DART-HRMS spectra besides those validated in the method. In order to evaluate the performance of the QuEChERS-DART-HRMS screening method in detecting the different analytes, we compared its results with those of the reference methods and calculated the resultant statistical indicators, expressed as overall accuracy, statistical sensitivity, and specificity. The overall accuracy is the number of correctly detected samples out of all the samples. While the specificity is a measure of the negative samples correctly identified by the method, the sensitivity indicates how well the method detects positive samples (Loong, 2003). Good agreement between QuEChERS-DART-HRMS and reference methods was achieved. The QuEChERS-DART-HRMS method achieved an overall accuracy of 89.6% (with 103/115 of the samples being correctly identified), a statistical sensitivity of 88.2%, and a statistical specificity of 91.5%. A confusion matrix reporting the results of the two methods’ comparison is reported in Supplementary Tables S3, S4 clearly shows the caveats of the technique by highlighting (in grey) the molecules that are more difficult to detect. Specifically, difficult detection was encountered for the rodenticides brodifacoum (not detected in 11/14 samples that actually contained this analyte), bromadiolone (5/7) (which are also those with lowest recovery and worst repeatability), and the chlorinated pesticide, endosulfan (8/10). This discrepancy is attributable to the LOD, which is higher in QuEChERS-DART-HRMS (500 μg/kg) than in conventional techniques (100 μg/kg). However, the difficulty of detecting the two rodenticides (brodifacoum and bromadiolone) was successfully overcome by detecting the bitter compound (denatonium benzoate), which is mandatorily added to rodenticide formulations. Specifically, among a total of 29 authentic samples containing coumaric rodenticides, we correctly detected denatonium benzoate in 29/29 samples. Five false positive were also encountered, thus bringing into question the specificity of the method for authentic samples. Considering only the poison baits, QuEChERS-DART-HRMS demonstrated an accuracy of 96.61%, with 57 out of 59 correctly identified, a sensitivity of 97.5%, and a specificity of 94.74% (Supplementary Table S4).

**TABLE 1:** Analytes detected in each authentic sample by QuEChERS-DART-HRMS and reference methods.

Sample No.	Type of sample	Analyte identified by reference methods	Analyte identified by QuEChERS-DART-HRMS
1	Gastrointestinal content	Brodifacoum	Brodifacoum (and DB)
2	Bait	Difenacoum	Difenacoum (and DB)
3	Bait	Flocoumafen	Flocoumafen (and DB)
4	Bait	Metaldehyde	Metaldehyde
5	Bait	Oxamyl	Oxamyl
6	Bait	Cyhalothrin and methiocarb	Cyhalothrin and methiocarb
7	Bait	Brodifacoum	Brodifacoum (and DB)
8	Gastrointestinal content	Brodifacoum	Brodifacoum (and DB)
9	Gastrointestinal content	Methomyl	Methomyl
10	Bait	Difenacoum	Difenacoum (and DB)
11	Gastrointestinal content	Methamidophos	Methamidophos
12	Gastrointestinal content	Metaldehyde	Metaldehyde
13	Bait	Metaldehyde	Metaldehyde
14	Bait	Metaldehyde	Metaldehyde
15	Bait	Brodifacoum	Brodifacoum (and DB)
16	Gastrointestinal content	Metaldehyde	Methiocarb
17	Bait	Flocoumafen	Flocoumafen (and DB)
18	Bait	Brodifacoum	Brodifacoum (and DB)
19	Bait	Metaldehyde	Endosulfan
20	Bait	Brodifacoum	Brodifacoum (and DB)
21	Bait	Metaldehyde	Bromadiolone (and DB)
22	Bait	Brodifacoum	n.d (only DB)
23	Gastrointestinal content	Metaldehyde	Brodifacoum (and DB)
24	Bait	Metaldehyde	Metaldehyde
25	Gastrointestinal content	Brodifacoum	Bendiocarb
26	Bait	Carbofuran	Carbofuran
27	Bait	Brodifacoum	Brodifacoum (and DB)
28	Bait	Metaldehyde	Metaldehyde
29	Bait	Brodifacoum	Brodifacoum (and DB)
30	Bait	Brodifacoum	Bromadiolone (and DB)
31	Bait	Difenacoum	Difenacoum (and DB)
32	Bait	Difenacoum	Difenacoum (and DB)
33	Gastrointestinal content	Brodifacoum	n.d (only DB)
34	Bait	Metaldehyde	Metaldehyde
35	Bait	Endosulfan	Endosulfan
36	Bait	Brodifacoum	Bromadiolone (and DB)
37	Bait	Difenacoum	Difenacoum (and DB)
38	Bait	Brodifacoum	Bromadiolone (and DB)
39	Bait	Brodifacoum	Bromadiolone (and DB)
40	Bait	Brodifacoum	Bromadiolone (and DB)
41	Gastrointestinal content	Metaldehyde	Metaldehyde
42	Bait	Coumatetralyl	Coumatetralyl
43	Gastrointestinal content	Metaldehyde	Metaldehyde
44	Bait	Difenacoum	Difenacoum (and DB)
45	Liver	n.d.	n.d.
46	Gastrointestinal content	n.d.	n.d.
47	Gastrointestinal content	n.d.	n.d.
48	Bait	n.d.	n.d.
49	Bait	n.d.	n.d.
50	Bait	Methomyl and aldicarb	Methomyl and aldicarb
51	Liver and gastrointestinal content	n.d.	Aldicarb
52	Bait	n.d.	n.d.
53	Gastrointestinal content	n.d.	n.d.
54	Liver	n.d.	n.d.
55	Gastrointestinal content	n.d.	n.d.
56	Bait	n.d.	n.d.
57	Bait	n.d.	coumafuryl
58	Bait	n.d.	n.d.
59	Bait	n.d.	n.d.
60	Bait	n.d.	n.d.
61	Gastrointestinal content	Metaldehyde	Metaldehyde
62	Gastrointestinal content	Metaldehyde	Metaldehyde
63	Liver	Bromadiolone	Bromadiolone (and DB)
64	Gastrointestinal content	n.d.	n.d.
65	Liver	Bromadiolone	n.d. (only DB)
66	Gastrointestinal content	Endosulfan	Endosulfan
67	Liver	n.d.	n.d.
68	Liver	n.d.	n.d.
69	Liver	n.d.	n.d.
70	Gastrointestinal content	n.d.	n.d.
71	Gastrointestinal content	n.d.	n.d.
72	Gastrointestinal content	n.d.	n.d.
73	Gastrointestinal content	Metaldehyde	Ethiofencarb and methiocarb
74	Bait	Metaldehyde	Ethiofencarb and methiocarb
75	Bait	n.d.	n.d.
76	Liver	n.d.	n.d.
77	Gastrointestinal content	Endosulfan	n.d.
78	Bait	Endosulfan	Endosulfan
79	Gastrointestinal content	n.d.	n.d.
80	Gastrointestinal content	n.d.	n.d.
81	Bait	n.d.	n.d.
82	Bait	n.d.	n.d.
83	Gastrointestinal content	n.d.	n.d.
84	Gastrointestinal content	Alfa and beta endosulfan	n.d.
85	Gastrointestinal content	n.d.	Warfarin
86	Gastrointestinal content	Alfa and beta endosulfan	Endosulfan
87	Gastrointestinal content	Metaldehyde	Metaldehyde
88	Gastrointestinal content	n.d.	n.d.
89	Gastrointestinal content	Alfa and beta endosulfan	Endosulfan
90	Gastrointestinal content	Alfa and beta endosulfan	Endosulfan
91	Liver	n.d.	n.d.
92	Bait	Alfa and beta endosulfan	Endosulfan
93	Liver	n.d.	n.d.
94	Gastrointestinal content	n.d.	n.d.
95	Liver	Bromadiolone	n.d. (only DB)
96	Liver	n.d	Metaldehyde
97	Bait	Metaldehyde + coumatetralyl	Metaldehyde
98	Bait	Metaldehyde	Metaldehyde
99	Liver	Oxamyl	Methomyl/oxamyl
100	Gastrointestinal content	Oxamyl	methomyl/oxamyl
101	Gastrointestinal content	n.d.	Ethiofencarb/methiocarb
102	Gastrointestinal content	n.d.	n.d.
103	Gastrointestinal content	n.d.	n.d.
104	Bait	n.d.	n.d.
105	Bait	n.d.	n.d.
106	Bait	n.d.	n.d.
107	Bait	Methomyl	Methomyl
108	Bait	n.d.	n.d.
109	Bait	n.d.	n.d.
110	Bait	n.d.	n.d.
111	Gastrointestinal content	n.d.	n.d.
112	Gastrointestinal content	n.d.	n.d.
113	Gastrointestinal content	Metaldehyde	n.d.
114	Bait	n.d.	n.d.
115	Bait	n.d.	n.d.

n.d., not detected; DB, denatonium benzoate.

**TABLE 2:** Other compounds detected in the 115 authentic samples from cases of suspected animal poisoning.

Class	Analyte	Formula	Type of ion	Observed *m/z*	Theoretical *m/z*	Error (ppm)
Tetroxocane	Metaldehyde	C_8_H_16_O_4 _	[M + NH_4_]^+^	195.1394	195.1387	3.6
Organochlorinate	Endosulfan	C_9_H_6_Cl_6_O_3_S	[M + NH_4_]^+^	421.8502	481.8507	−1
Organophosphate	Methamidophos	C_2_H_8_NO_2_PS	[M + H]^+^	142.0086	142.0086	0
Bitter agent	Denatonium benzoate	C_28_H_34_N_2_O_3_	[M-C7H6O2]^+^	325.226	325.2258	−0.6

## Discussion

According to the results described above, this ambient mass spectrometry method can be recommended for the screening of baits collected in the investigation of suspected animal poisoning cases. Specifically, we assessed the method’s performance in detecting xx compounds (analytes) by comparing the results with those of the recognized reference methods, as was done previously in clinical diagnosis (Eberlin et al., 2013; Eberlin et al.) and food safety analysis (Schurek et al., 2008). The comparison of measurements obtained by a new technique with measurements produced by an established method is mandatory to see whether they agree sufficiently for the new to replace or support the established method. Based on this, our QuEChERS-DART-HRMS method showed extremely promising results in the analysis of baits, and this was likely due to the high content of poison present in the authentic field samples used.

As recently reported by Gallocchio *et al.* (Gallocchio; Moressa; Stella; Rosin et al., 2021), the minimum amount of toxic compounds found in baits is much higher than our LOD. Specifically, Gallocchio et al reported the minimum and maximum amount of toxic compounds quantified in more than 800 authentic samples (including liver, gastrointestinal content, and baits) collected from April 2019 to October 2020 by our institute. In baits, the vast majority of these toxic compounds ranged in concentrations between thousands of µg/kg and hundreds of thousands µg/kg. Three exceptions were brodifacoum, bromadiolone, and aldicarb, for which the minimum amounts reported were between 100 and 244 μg/kg (maximum concentrations observed were hundreds of thousands of µg/kg) (Gallocchio; Moressa; Stella; Rosin et al., 2021). These three compounds are also those presenting lower identification rates (Supplementary Table S5) and lower recoveries (Supplementary Table S2 **)** in our study**.** Due to the relatively high LODs for these three compounds, characteristic of the novel QuEChERS-DART-HRMS method compared to the traditional ones, there is greater difficulty in identifying these three analytes. We stress though, that this is compensated by the significant reduction in the time required for sample preparation and related costs. Moreover, the difficulty of detecting the two rodenticides was successfully overcome by looking for the bitter compound (denatonium benzoate), mandatorily added to rodenticide products. Note that the baits marketed for rodent control must contain a bittering agent to make these products unpalatable to children, thus reducing the likelihood bait will be eaten. Therefore, the presence of the bitter agent can suggest the presence of rodenticide in the investigated sample.

One of the reported caveats of ambient mass spectrometry is the strong matrix effect (that results in the low recoveries reported in the SI), which can lead to fluctuations in ion abundance. Detection of a compound by DART-HRMS could depend on the matrix of the baits, e.g., whether it is done with suasages, meat, fat, bread. Although, this results in limited quantification abilities, this issue can be disgarded when screening applications are envised. In this study, the matrix effect did not affect our profiling analysis, as was demonstrated by our CV% values. Note that CV% analysis is routinely used to assess data reproducibility in analytical sciences, and it has also been used in ambient mass spectrometry studies for the same purpose (Dill et al., 2011; Abbassi-Ghadi et al., 2015; Bilkey et al., 2016; Riuzzi et al., 2021; Tata et al., 2022b). In the present study, we have shown once more that QuEChERS-DART-HRMS is characterized by a CV% lower than 20%, which is in line with United States Food and Drug Administration guidelines for chromatographic analytical techniques (Guidance, 2013). The low CV% values indicated an acceptable level of precision and theoretical scope for screening targeted molecules by QuEChERS-DART-HRMS, as was similarly reported by Shureck et al. (Schurek et al., 2008). These acceptable CV% values were achieved by utilizing the QuEChERS clean-up to minimize the notorious matrix effect associated with the DART-HRMS technique [extensively described by Gross et al. (2014)]. Although several studies applied QuEChERS as a sample clean-up prior to HPLC analysis of complex matrices, to the best of our knowledge, only two studies have combined QuEChERS with DART-HRMS (Hakami et al., 2021; Martínez-Villalba et al., 2013). Through use of the integrated QuEChERS-DART-HRMS approach described in our current study, anywhere between 20 samples can be extracted, purified, and analyzed by DART-HRMS (in triplicate) in 2 h, with minimal training needed for user to become proficient. In contrast, the LC and GC run-times (in combination with the QuEChERS extraction method) for the clean-up, separation and detection would take between 1.5 and 2 h per sample. Within that time-frame, one analyst could screen 20 suspect samples using integrated QuEChERS-DART-HRMS.

Note that during the autopsy or the inspection of the collected baits, the high solubility of some toxic compounds, such as some pesticides and rodenticides, prevents the veterinary pathologists from understanding which specific classes of toxins must be investigated by the analytical laboratory. This leads to requests from the veterinary pathologists for all possible toxicological analyses to be conducted on each sample, with the consequent employment of multiple chromatographic-based methods on each sample. By using QuEChERS-DART-HRMS to screen samples, substantial amounts of time can be saved, even with the negative samples still being included in confirmatory analysis testing rotation. Moreover, QuEChERS-DART-HRMS favors great reductions in the use of solvents and other chromatography consumables, which reduces costs and lessens risks to human and environmental health. For these reasons, we now propose all bait samples from suspect cases of animal poisoning arriving at our institute are initially screened by the novel QuEChERS-DART-HRMS technique, with the positive results of screening being further confirmed by the appropriate reference method(s), and only those samples with negative screening results being re-analyzed by all the reference methods.

## Conclusion

In this study we used the combined QuEChERS-DART-HRMS approach to conduct initial screening of baits from cases of suspected animal poisoning. While the approach is not confirmatory, as it does not enable the discrimination of toxicants at amounts lower than 500 μg/kg, this caveat is offset by the analysis of samples in seconds without instrument contamination or compound carryover. Therefore, when implemented as an orthogonal method for preliminary screening of suspect poison baits, this novel approach has great potential to guide the choice of reference methods, assist the toxicology laboratories by facilitating the redeployment GC-MS and LC-MS instruments, thereby reducing costs and increasing efficiency. Attempts to combine DART-HRMS with solid phase microextraction (SPME) for ultrafast enrichment of toxic compounds to achieve lower limits of detection (Gómez-Ríos et al., 2017; Vasiljevic and Pawliszyn, 2019) are ongoing in our laboratories.

## Data Availability

The original contributions presented in the study are included in the article/Supplementary Material, further inquiries can be directed to the corresponding author.
