# Supplementary material for: Rapid, novel screening of toxicants in poison baits, and autopsy specimens by ambient mass spectrometry
Source: Front Chem. 2022 Aug 25;10:982377. doi: 10.3389/fchem.2022.982377 (PMC9452653; doi:10.3389/fchem.2022.982377)
Supplement: Supplementary file 1 [file DataSheet1.docx]

Rapid, novel screening of toxicants in poison baits and autopsy specimens by ambient mass spectrometry.

Alessandra Tata^1*^, , Ivana Pallante^2^, Carmela Zacometti^1^, Alessandra Moressa^3^, Marco Bragolusi^1^, Alessandro Negro^1^, Andrea Massaro^1^, Giovanni Binato^3^, Federica Gallocchio^3^, Roberto Angeletti, Nicola Pozzato^2^, Roberto Piro^1^.

**Supporting information**

**Figure S1. Overlapped DART-HRMS spectra of the azinphos-ethyl at 5 mg/kg. In violet the signal of the azinphos-ethyl standard diluted in acetone. The red signal corresponds to the spiked azinphos-ethyl (at 5 mg/kg ) in a sausage extracted by QuEChERS. In yellow, the signal of azinphos-ethyl spiked in the sausage with a concentration of 5 mg/kg and extracted with pure acetonitrile. In green the signal of the blank.**

**Table S1. List of certified toxic compounds detected by QuEChERS-DART-HRMS in spiked sausages. The observed and theoretical *m/z*, error (ppm), elemental formula, type of ion, limit of detection (LOD), and repeatability (CV%) at the LOD and at higher concentration are reported.**

| **Class** | **Analyte** | **Formula** | **Type of ion** | **observed *m/z*** | **Theor** | **Error** | **LOD**  **µg/kg** | **CV% at 500 µg/kg** | **CV% at 5000 µg/kg** |
| --- | --- | --- | --- | --- | --- | --- | --- | --- | --- |
|  |  |  |  |  | ***m/z*** | **ppm** |  |  |  |
| **Carbamates** | Aldicarb | C_7_H_14_N_2_O_2_S | [M+NH_4_]^+^ | 208.1114 | 208.1114 | 0 | 500 | 8.66% | 4.03% |
|  | Bendiocarb | C_11_H_16_N_3_O_3_PS_3_ | [M+H]^+^ | 224.0917 | 224.0917 | 0 | 500 | 43.96% | 14.91% |
|  | Benfuracarb | C_11_H_13_NO_4_ | [M+H]^+^ | 411.1941 | 411.1948 | -1.6 | 500 | 0.00% | 8.91% |
|  | Carbaryl | C_12_H_11_NO_2_ | [M+H]^+^ | 202.0859 | 202.0863 | -1.8 | 500 | 59.21% | 24.75% |
|  | Carbofuran | C_12_H_15_NO_3_ | [M+H]^+^ | 222.1121 | 222.1125 | -1.7 | 500 | 19.89% | 16.66% |
|  | Carbosulfan | C_20_H_32_N_2_O_3_S | [M+H]^+^ | 381.2199 | 381.2206 | -1.7 | 500 | 21.81% | 6.94% |
|  | Ethiofencarb/Methiocarb | C_11_H_15_NO_2_S | [M+H]^+^ | 226.0891 | 226.0896 | -2.2 | 500 | 33.33% | 8.66% |
|  | Furathioncarb | C1_8_H_26_N_2_O_5_S | [M+H]^+^ | 383.1628 | 383.1635 | -1.8 | 500 | 15.81% | 18.39% |
|  | Methomyl | C_5_H_10_N_2_O_2_S | [M+H]^+^ | 162.046 | 162.0463 | -1.8 | 500 | 22.93% | 7.91% |
|  | Oxamyl | C_7_H1_3_N_3_O_3_S | [M+H]^+^ | 220.0745 | 220.075 | -2.2 | 500 | 7.16% | 9.92% |
|  | Pirimicarb | C_11_H_18_N_4_O_2_ | [M+H]^+^ | 239.1497 | 239.1503 | -2.2 | 500 | 4.71% | 14.78% |
|  | Propoxur | C_11_H_15_NO_3_ | [M+H]^+^ | 210.1121 | 210.1125 | -2.1 | 500 | 11.78% | 11.95% |
| **Anticoagulant rodonticides** | Brodifacoum | C_31_H_23_BrO_3_ | [M+H]^+^ | 523.0895 | 523.0903 | -1.5 | 500 | 49.49% | 13.56% |
|  | Bromadiolone | C_30_H_23_BrO_4_ | [M-H_2_O+H]^+^ | 509.0741 | 509.0747 | -1.2 | 500 | 16.67% | 7.91% |
|  | Coumachlor | C_19_H_15_ClO_4_ | [M+H]^+^ | 343.0717 | 343.0732 | -5 | 500 | 19.37% | 18.18% |
|  | Coumafuryl | C_17_H_14_O_5_ | [M+H]^+^ | 299.0899 | 299.0914 | -4.8 | 500 | 15.52% | 11.44% |
|  | Coumatetralyl | C_19_H_16_O_3_ | [M+H]^+^ | 293.1171 | 293.1172 | -0.3 | 500 | 11.90% | 11.16% |
|  | Difenacoum | C_31_H_24_O_3_ | [M+H]^+^ | 445.1793 | 445.1798 | -1.1 | 500 | 33.72% | 16.57% |
|  | Diphenadione | C_23_H_16_O_3_ | [M+H]^+^ | 341.1156 | 341.1172 | -4.7 | 500 | 14.29% | 13.02% |
|  | Flocoumafen | C_33_H_25_F_3_O_4_ | [M+H]^+^ | 543.1772 | 543.1778 | -1.1 | 500 | 13.19% | 12.68% |
|  | Strychnine | C_21_H_22_N_2_O_2_ | [M+H]^+^ | 335.1754 | 335.1742 | 3.5 | 500 | 11.35% | 15.32% |
|  | Pindone | C_14_H_14_O_3_ | [M+H]^+^ | 231.1007 | 231.1016 | -3.9 | 500 | 17.63% | 18.26% |
|  | Warfarin | C_19_H_16_O_4_ | [M+H]+ | 309.1122 | 309.1116 | 1.9 | 500 | 17.34% | 9.83% |
| **organophosphates insecticides** | Azinphos-ethyl | C_12_H_16_N_3_O_3_PS_2_ | [M+H]^+^ | 346.0441 | 346.0443 | -0.6 | 500 | 11.24% | 8.33% |
|  | Disulfoton | C_8_H_19_O_2_PS_3_ | [M+H]^+^ | 275.0352 | 275.0358 | -1.8 | 5000 | 0.00% | 11.64% |
|  | Malathion | C_10_H_19_O_6_PS_2_ | [M+H]^+^ | 331.0428 | 331.0433 | -3.1 | 500 | 17.35% | 20.30% |
|  | Parathion | C_10_H_14_NO_5_PS | [M+H]^+^ | 292.0396 | 292.0403 | -2.5 | 5000 | 5.97% | 5.97% |
|  | Parathion-methyl | C_8_H_10_NO_5_PS | [M+H]^+^ | 264.0085 | 264.009 | -1.9 | 5000 | 13.10% | 13.10% |
|  | Phosmet | C_11_H_12_NO_4_PS_2_ | [M+H]^+^ | 318.0017 | 318.0018 | -2.8 | 500 | 18.70% | 15.61% |
|  | Phoxim | C_12_H_15_N_2_O_3_PS | [M+H]^+^ | 299.061 | 299.0614 | -0.3 | 5000 | 13.35% | 13.35% |
|  | Profenofos | C_11_H_15_BrClO_3_PS | [M+H]^+^ | 372.9416 | 372.9414 | 0.5 | 500 | 18.37% | 2.85% |
| **pyretroids** | Cyfluthrin | C_22_H_18_Cl_2_FNO_3_ | [M+NH_4_]^+^ | 451.0979 | 451.0986 | -1.55 | 500 | 10.74% | 9.08% |
|  | Cyhalothrin | C_23_H_19_ClF_3_NO_3_ | [M+NH_4_]^+^ | 467.1339 | 467.1344 | -1 | 500 | 16.88% | 11.42% |
|  | Cypermethrin | C_22_H_19_Cl_2_NO_3_ | [M+NH_4_]^+^ | 433.1076 | 433.108 | -0.9 | 500 | 7.69% | 8.69% |
|  | Deltamethrin | C_22_H_19_Br_2_NO_3_ | [M+NH_4_]^+^ | 521.0065 | 521.007 | -0.9 | 500 | 9.44% | 7.89% |
|  | Fenvalerate | C_25_H_22_ClNO_3_ | [M+NH_4_]^+^ | 437.1621 | 437.1626 | -1.1 | 500 | 8.11% | 12.32% |
|  | Permethrin | C_21_H_20_Cl_2_NO_3_ | [M+NH_4_]^+^ | 408.1123 | 408.1128 | 1.2 | 500 | 15.22% | 14.95% |

**CV% was calculated on signal intensities of the analytes for the three instrumental replicates.*

**Table S2. Recoveries of toxic compounds spiked at concentration of 5000 µg/kg in sausage**

| **Class** | **Analyte** | **RECOVERY** |
| --- | --- | --- |
|  |  |  |
| **Carbamates** | Aldicarb | 28% |
|  | Bendiocarb | 21% |
|  | Benfuracarb | 46% |
|  | Carbaryl | 44% |
|  | Carbofuran | 45% |
|  | Carbosulfan | 7% |
|  | Ethiofencarb/Methiocarb | 40% |
|  | Furathioncarb | 6% |
|  | Methomyl | 13% |
|  | Oxamyl | 13% |
|  | Pirimicarb | 22% |
|  | Propoxur | 48% |
| **Anticoagulant rodonticides** | Brodifacoum | 8% |
|  | Bromadiolone | 27% |
|  | Coumachlor | 22% |
|  | Coumafuryl | 13% |
|  | Coumatetralyl | 19% |
|  | Difenacoum | 15% |
|  | Diphenadione | 22% |
|  | Flocoumafen | 67% |
|  | Pindone | 77% |
|  | Warfarin | 22% |
| **organophosphates insecticides** | Azinphos-ethyl | 25% |
|  | Disulfoton | 88% |
|  | Malathion | 52% |
|  | Parathion | 12% |
|  | Parathion-methyl | 10% |
|  | Phosmet | 15% |
|  | Phoxim | 9% |
|  | Profenofos | 19% |
| **pyretroids** | Cyfluthrin | 24% |
|  | Cyhalothrin | 22% |
|  | Cypermethrin | 19% |
|  | Deltamethrin | 16% |
|  | Fenvalerate | 27% |
|  | Permethrin | 12% |
|  | Strychnine | 10% |
|  |  |  |

**Figure S2. Overlapped DART-HRMS spectra of Methamidophos at 5 mg/kg extracted from spiked sample (blue) and from a real autoptic specimen (pink).**

**Table S3. Confusion matrix with the results obtained by official methods and QUECHERS-DART-HRMS screening of authentic samples.**

|  |  | *actual* | |
| --- | --- | --- | --- |
|  |  | **Poison baits**  **by reference methods** | **Negative samples**  **by reference methods** |
| *predicted* | **Poison baits**  **by DART-HRMS** | 60 | 4 |
|  | **Negative baits**  **by DART-HRMS** | 8 | 43 |

**Table S4. Confusion matrix with the results obtained by official methods and DART-HRMS screening for baits only.**

|  |  | *actual* | |
| --- | --- | --- | --- |
|  |  | **Poison baits**  **by reference methods** | **Negative samples**  **by reference methods** |
| *predicted* | **Poison baits**  **by DART-HRMS** | 39 | 1 |
|  | **Negative baits**  **by dart hrms** | 1 | 18 |

**Table S5. Comparison between reference methods results and DART-HRMS data**

| **TOXIC COMPOUND** | **TYPE OF SAMPLE** | **IDENTIFICATION BY REFERENCE METHODS** | **IDENTIFICATION BY DART-HRMS** |
| --- | --- | --- | --- |
| Bendiocarb | gastrointestinal contents | 1 | 1 |
| Brodifacoum | gastrointestinal contents, liver, baits | 14 | 11 |
| Bromadiolon | gastrointestinal contents and baits | 7 | 5 |
| Carbofuran | gastrointestinal contents and baits | 1 | 1 |
| Coumatetralyl | baits | 1 | 1 |
| Cyalothrin + Methiocarb | baits | 1 | 1 |
| Difenacoum | baits | 6 | 6 |
| Endosulfan | gastrointestinal contents and baits | 10 | 8 |
| Flocoumafen | baits | 2 | 2 |
| Metaldehyde | gastrointestinal contents and baits | 14 | 13 |
| Metaldehyde and Coumatetralyl | bait | 1 | 0 |
| Methamidofos | gastrointestinal contents | 1 | 1 |
| Methiocarb | gastrointestinal contents and baits | 3 | 1 |
| Methomyl | gastrointestinal contents and baits | 2 | 2 |
| Methomyl and Aldicarb | baits | 1 | 1 |
| Oxamyl | gastrointestinal contents liver and baits | 3 | 3 |
| Negative Results | gastrointestinal contents baits | 43 | 51 |
